# Involvement of Prohibitin Upregulation in Abrin-Triggered Apoptosis

**DOI:** 10.1155/2012/605154

**Published:** 2011-09-25

**Authors:** Yu-Huei Liu, Konan Peck, Jung-Yaw Lin

**Affiliations:** ^1^Institute of Biochemistry and Molecular Biology, College of Medicine, National Taiwan University, Taipei 100, Taiwan; ^2^Graduate Institute of Integrated Medicine of Chinese Medicine, China Medical University, Taichung 404, Taiwan; ^3^Department of Medical Genetics and Medical Research, China Medical University Hospital, Taichung 404, Taiwan; ^4^Institute of Biomedical Sciences, Academia Sinica, Taipei 115, Taiwan

## Abstract

Abrin (ABR), a protein purified from the seeds of *Abrus precatorius*, induces apoptosis in various types of cancer cells. However, the detailed mechanism remains largely uncharacterized. By using a cDNA microarray platform, we determined that prohibitin (PHB), a tumor suppressor protein, is significantly upregulated in ABR-triggered apoptosis. ABR-induced upregulation of PHB is mediated by the stress-activated protein kinase/c-Jun NH2-terminal kinase (SAPK/JNK) pathway, as demonstrated by chemical inhibitors. In addition, ABR significantly induced the expression of Bax as well as the activation of caspase-3 and poly(ADP-ribose) polymerase (PARP) in Jurkat T cells, whereas the reduction of PHB by specific RNA interference delayed ABR-triggered apoptosis through the proapoptotic genes examined. Moreover, our results also indicated that nuclear translocation of the PHB-p53 complex may play a role in the transcription of Bax. Collectively, our data show that PHB plays a role in ABR-induced apoptosis, which may be helpful for the development of diagnostic or therapeutic agents.

## 1. Introduction

Abrin (ABR), purified from the seeds of *Abrus precatorius*, belongs to the family of type II ribosome-inactivating proteins (RIPs) that contain 2 subunits. These include a toxic A chain with RNA *N*-glycosidase activity and a galactose-binding B chain with lectin activity [1]. Like ricin from *Ricinus communis*, the A chain of ABR functions via the inhibition of protein biosynthesis through depurination of a single adenine residue (A^4324^) of the 28S ribosomal RNA [2, 3]. In contrast, the B chain of ABR functions by interacting with the galactose moiety of glycoproteins or glycolipids on the cell membrane and is internalized into cells through receptor-mediated endocytosis. Several reports have documented that ABR is mitogenic [4], antifertility [5], antitumoral [6, 7], and immunopotentiating [8–12] agent. In addition to its ability to inhibit protein synthesis, ABR is believed to adopt alternative mechanisms to trigger cell apoptosis [13]; despite this, it is relatively less toxic to normal cells than to cancer cells [10, 14]. Our previous studies have implied that apoptosis induced by ABR could be partially independent of its RNA *N*-glycosidase activity and instead be mediated by its binding and the decrease of antioxidant protein-1 (AOP-1), increase of reactive oxygen species production, and release of cytochrome c into the cytosol [15]. 

Prohibitin (PHB) is localized on the cell membrane, mitochondria, and nucleus; this localization may play a pivotal role in its regulation of cell-cycle progression by the inhibition of DNA replication in multiple cell types [16]. The protumorigenic versus antitumorigenic role of PHB in cancer cells remains controversial. An oncogenic role has been identified for PHB in different kinds of cancer cells, including those of the breast [17], bladder [18], gastric [19], ovary [20], and prostate [21], whereas PHB's role as a tumor suppressor has been demonstrated in esophageal squamous cell carcinoma [22–26]. These opposing effects of PHB in cancer may be due to 2 possible mechanisms. One is a polymorphism in PHB [27]. The other involves its subcellular localization; increased levels of PHB on the cell membrane facilitates tumorigenesis through its interaction with c-Raf induced by the Ras oncogene [28], whereas increased levels of PHB in the nucleus induces apoptosis by increasing the transcriptional activity of p53 and its translocation to the cytoplasm [29]. 

In order to understand the genetic basis of the apoptotic signaling exerted by ABR, a microarray platform was used to investigate the expression profiles of genes in Jurkat T cells after ABR exposure. Among the genes identified, PHB was significantly upregulated; however, it has yet to be determined whether PHB plays a role in ABR-triggered apoptosis. Here, we report that overexpression of PHB is involved in ABR-triggered Jurkat T cell apoptosis. Upregulation of PHB through the JNK/SAPK pathway activated the pro-apoptotic gene Bax via the accumulation and translocation of the PHB-p53 complex to the cytoplasm. The elucidation of the changes in gene expression and the cellular mechanisms of the response to ABR exposure may be helpful for the development of diagnostic or therapeutic agents.

## 2. Materials and Methods

### 2.1. Isolation of ABR

ABR was isolated from seeds of the red variety of *A. precatorius* using Sepharose 6B affinity column chromatography and purified as described in a previous study [30]. The purity and molecular weight of ABR protein were confirmed by Coomassie blue staining (data not shown). The stock protein solution was diluted with phosphate-buffered saline (PBS, pH 7.2) to a concentration of 100 *μ*M. 

### 2.2. Cells and Culture Conditions

The human Jurkat T leukemia cancer cell line was obtained from American Type Culture Collection and maintained in RPMI-1640 medium supplemented with 10% heat-inactivated fetal bovine serum, 100 U/mL penicillin, and 100 *μ*g/mL streptomycin. Cells were grown in suspension in a 5% CO_2_ humidified atmosphere at 37°C.

### 2.3. Cell Proliferation Assay

Cells were seeded in 96-well plates at a density of 5 × 10^3^ cells per well and were treated with (various doses) or without ABR. At the indicated times, viable cells were analyzed by measuring the conversion of the tetrazolium salt 4-[3-(4-iodophenyl)-2-(4-nitrophenyl)-2H-5-tetrazolio]-1,3-benzene disulfonate (WST-1) to formazan. The formazan dye produced by metabolically active cells was measured with a scanning multiwell spectrophotometer after 4 h of incubation, according to the manufacturer's instructions.

### 2.4. The cDNA Microarray System

The gene expression patterns regulated by ABR were analyzed with a previously established cDNA microarray platform (9600 probes) [31]. The mRNA from cells with or without ABR treatment was extracted using an Oligotex-dT column (Qiagen). A 2 mg quantity of each mRNA sample was labeled with biotin or digoxigenin for membrane hybridization, dual-color detection, and image analysis as described previously [31]. 

### 2.5. Antibodies and Chemical Inhibitors

An antibody specific to PHB was purchased from Lab Vision Corporation. An antibody specific to p53 was obtained from Santa Cruz Biotechnology. Antibodies specific to cleaved-caspase-3, and cleaved-poly(ADP-ribose) polymerase (PARP) were purchased from Cell Signaling. Antibodies against Bax and actin were obtained from Chemicon. The chemical inhibitors PD 98059, SB 203580, and SP600125 were purchased from Sigma. 

### 2.6. Immunoblotting

Total cell lysates were collected in lysis buffer (50 mM HEPES-KOH, pH 7.5, 1% Triton X-100, 150 mM NaCl, and protease inhibitor cocktail (Roche)). The extracts were centrifuged at 14000 rpm for 20 min, and then the clear supernatant was separated by using 10% SDS-PAGE. After transferred, the separated proteins to a polyvinyldene fluoride (PVDF) membrane (Immunobilon-P, 0.45 mm; Millipore, Billerica, Mass, USA) by using the NA-1512 semi-dry transfer apparatus (NIHON EIDO), the membranes were blocked with 5% skim milk in trisbuffered saline containing 1% Tween 20 (TBST, pH 7.4) at room temperature for 30 min and then incubated overnight at 4°C with primary antibodies. The membranes were washed 4 times with TBST for 10 min each at room temperature and incubated with HRP-conjugated secondary antibodies for 1 h at room temperature. The membranes were then washed 4 times with TBST. The proteins were visualized using the SuperSignal West Femto Chemiluminescent Kit (Thermo Scientific) and exposed to an X-ray film (Kodak). The Image J program (http://rsb.info.nih.gov/) was used for quantization the expression fold. For western blot analysis, the fold increase of the indicated proteins was determined by normalizing to corresponding actin expression. For IP-western analysis, the densitometry readings of the bands were normalized to control.

### 2.7. Enzyme-Linked Immunosorbent Assay (ELISA) for Phosphor MAPK Detection

Cells were treated with or without ABR after pretreating with the indicated inhibitors or vehicle (DMSO) alone. After the indicated period of time, cells were harvested and lysed as above. The PathScan MAP Kinase Multi-Target Sandwich ELISA kit was used to determine phosphor ERK, -p38, and-JNK/SAPK levels according to manufacturer's instruction (Cell Signaling).

### 2.8. Short Interfering RNA 

Short interfering RNAs (siRNAs) against PHB (sc-37629) and the negative control siRNA (sc-37007) were obtained from Santa Cruz Biotechnology. A total of 2 × 10^5^ cells were plated in a 6-well plate for 24 h, and siRNA transfection was carried out using the Lipofectamine 2000 kit according to the manufacturer's instructions (Invitrogen).

### 2.9. Terminal Deoxynucleotidyl Transferase-Catalyzed Deoxyuridine Triphosphate (dUTP)-Nick End Labeling (TUNEL) Method [15]

Apoptotic cell death was examined by TUNEL method as manufactory's suggestion (Roche Molecular Biochemicals). Each sample with 1 × 10^4^ events was analyzed with a Becton-Dickinson FACSCalibur, and the distribution of cells was determined.

### 2.10. Chromatin Immunoprecipitation (ChIP) Assays

Cells treated with or without ABR for the indicated time periods were examined. After fixing the protein-DNA complex using formaldehyde (1% final concentration) at room temperature for 10 min, the reaction was stopped with glycine. After washed and lysed the cells, the lysates were sonicated and centrifuged, and the supernatants were used for immunoprecipitation of Bax with PHB antibody or control IgG. Antibody-bound protein/DNA complexes were precipitated and eluted in 300 *μ*L of elution buffer (1% SDS, 50 mM NaHCO_3_). Cross-linking was reversed by heating at 65°C for 4 h. The DNA was resuspended in 200 *μ*L of distilled water and treated with 30 *μ*g of proteinase K at 37°C for 1 h, followed by phenol/chloroform extraction and ethanol precipitation. PCR was conducted using 100 ng of DNA as the template. The following PCR primers were used for the Bax promoter: forward primer 5′- CCGGGAATTCCAGACTGCA -3′ and reverse primer 5′- AGCTCTCCCCAGCGCAGAA -3′. Each band was quantitatively determined using the Image J program (http://rsb.info.nih.gov/). The densitometry readings of the bands were normalized to the input.

### 2.11. Statistical Analysis

SPSS 12.0 for Windows (SPSS Inc.) was used to analyze the data. A two-tailed paired-samples Student's *t*-test was used for statistical analysis of the comparative data from the two groups. *P*-value <0.05 values were considered statistically significant.

## 3. Results

### 3.1. ABR Induces Upregulation of PHB in Human Jurkat T Cells

**Figure 1:**
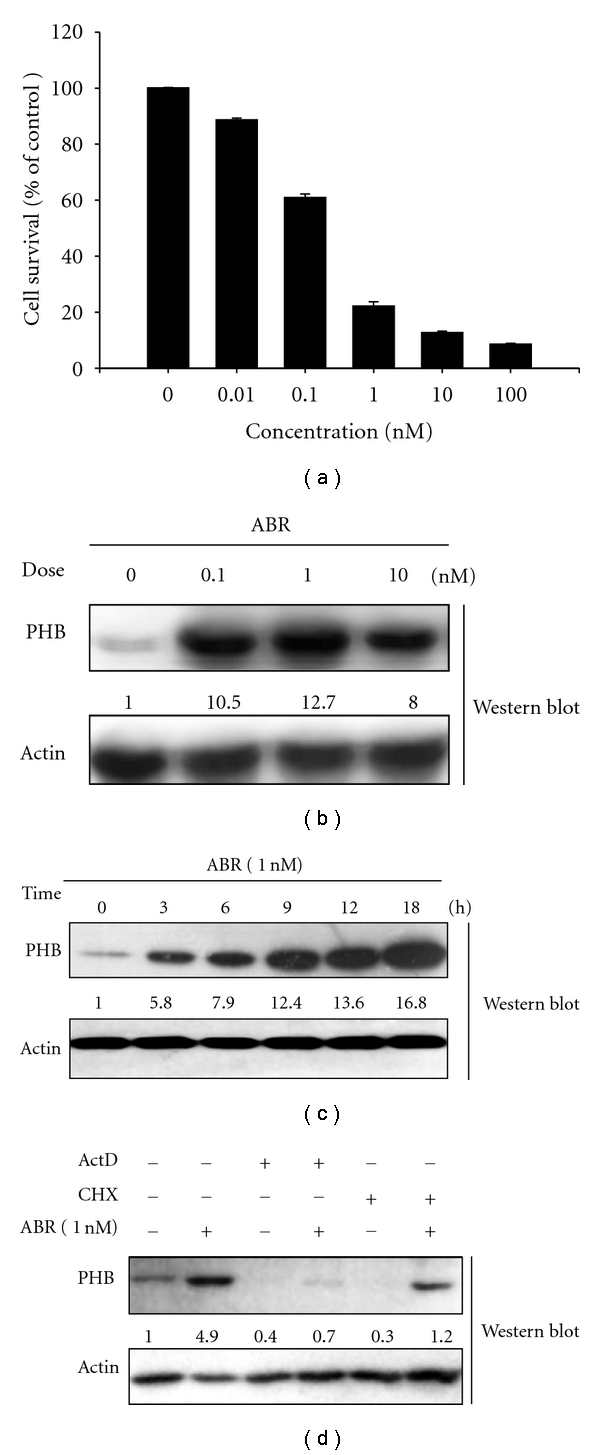
Abrin (ABR) upregulates prohibitin (PHB) expression through transcriptional regulation in Jurkat T cells. (a) ABR-induced cytotoxic activity in a dose-dependent manner in Jurkat T cells after 24 h treatment. The data are represented as mean ± SD from 3 independent experiments. (b) ABR (0.1–10 nM) significantly increased the expression of PHB after treatment for 9 h. (c) ABR (1 nM)-induced upregulation of PHB in a time-dependent manner. (d) ABR-induced PHB upregulation requires de novo RNA synthesis.

To evaluate the effect of ABR on leukemia cells in vitro, Jurkat T leukemia cells were exposed to 0.01–100 nM of ABR for 24 h. The cell viability was then determined by WST-1 assay. As shown in Figure 1(a), the growth of Jurkat T cells was reduced by ABR in a dose-dependent manner. The value of the 50% cytotoxic concentration (CC_50_) for the 24 h treatment was determined for the water fraction to be 0.32 ± 0.06 nM. The data are represented as mean ± SD from 3 independent experiments. 

Microarray analysis was used to identify novel candidates that are differentially expressed after 1 nM ABR treatment for 3 h. A total of 128 genes, out of the 9600 probes tested, were significantly altered by >1.5-fold in response to ABR (*P* < 0.05). The top 10 significant up-/down-regulated genes are listed in Table 1, sorted by fold increase or decrease. PHB, a significantly upregulated gene with diverse cellular functions, was selected for further investigation. To explore the potential role of PHB in ABR-treated apoptosis, Jurkat T cells were first treated with ABR (0.1–10 nM). As shown in Figure 1(b), ABR significantly increased the expression of PHB protein after treatment for 9 h. Cells were further treated with 1 nM ABR for different time periods. An initial increase of PHB protein was observed at the 3 h time point and was sustained for up to 18 h after ABR treatment (Figure 1(c)). To determine whether PHB upregulation due to ABR is because of increased transcription or increased RNA stability, the RNA synthesis inhibitor actinomycin D or the protein synthesis inhibitor cyclohexamide was preincubated with cells for 1 h before ABR was added. The results show that not only actinomycin D but also cyclohexamide significantly diminished ABR-induced PHB upregulation (Figure 1(d)). This finding suggests that ABR-induced PHB upregulation requires de novo RNA synthesis.

### 3.2. ABR Upregulates PHB Expression through the SAPK/JNK Pathway

**Figure 2:**
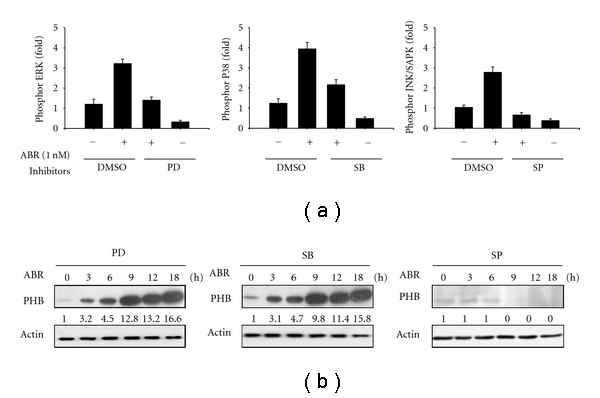
The JNK/SAPK signaling pathway is required for abrin (ABR)-triggered upregulation of prohibitin (PHB). (a) Cells were treated with or without indicated inhibitors for 1 h before ABR treatment. Effects of PD98059 (PD; 20 *μ*M), SB203580 (SB; 20 *μ*M), or SP600125 (SP; 30 *μ*M) on their target signaling molecules were shown. (b) Cells were pretreated with 20 *μ*M PD, 20 *μ*M SB, or 30 *μ*M SP for 1 h before ABR treatment. After the indicated period of time, only SP significantly inhibited the upregulation of PHB by ABR as shown by western blot analysis.

To explore which of the signaling pathways are required for ABR-induced upregulation of the PHB gene, several specific chemical inhibitors were used. The effect of these inhibitors was examined using an ELISA-based detection system (Figure 2(a)). Jurkat T cells were pretreated with PD98059 (PD, MEK inhibitor; 20 *μ*M), SB203580 (SB, p38 MAPK inhibitor; 20 *μ*M), or SP600125 (SP, JNK/SAPK inhibitor; 30 *μ*M) for 1 h, followed by treatment with ABR for the time indicated; total protein was used to determine PHB expression. SP significantly reduced the ABR-induced PHB expression, whereas the 2 other kinase inhibitors PD and SB rarely affected on the upregulation of PHB (Figure 2(b)). These results suggest the possible involvement of JNK/SAPK, but not of ERK1/2 or p38 MAPK, in the regulation of PHB expression in Jurkat T cells treated with ABR.

### 3.3. PHB Is Involved in ABR-Induced Cell Apoptosis

**Figure 3:**
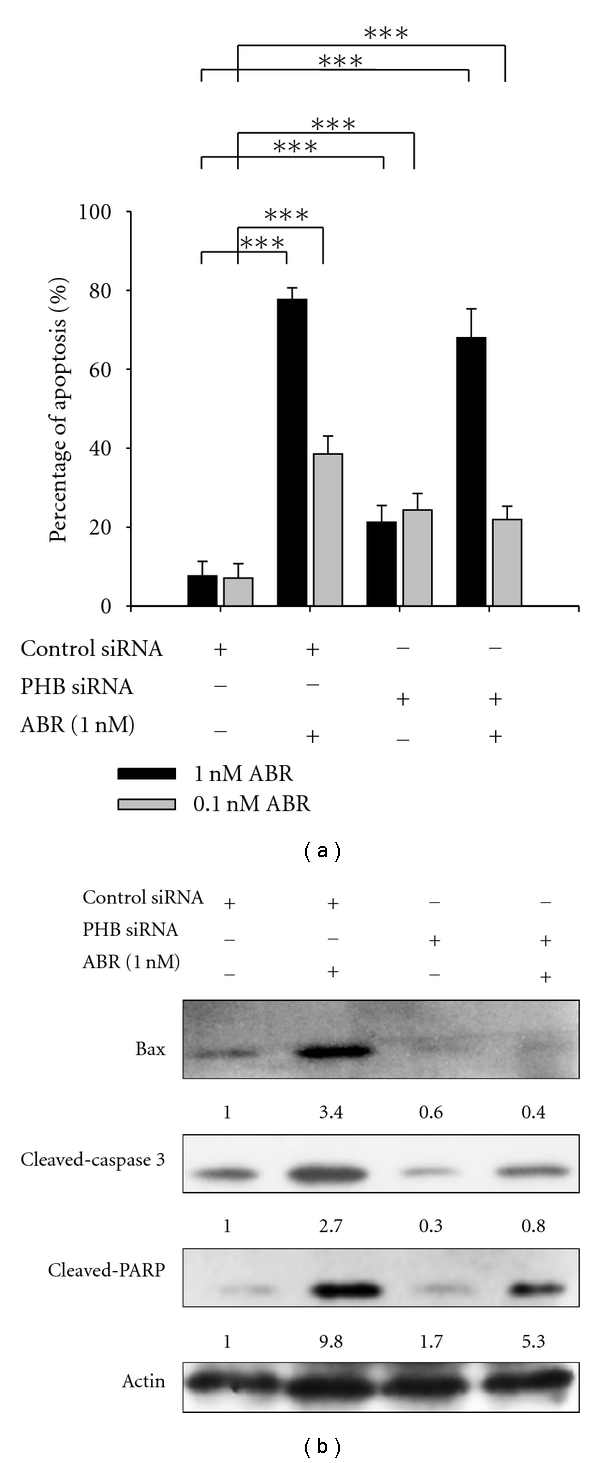
Downregulation of prohibitin (PHB) delays abrin (ABR)-triggered cell apoptosis. (a) Downregulation of PHB expression with siRNA delays ABR-triggered cell apoptosis in Jurkat T cells. The cells were treated with 1 nM ABR for 18 h (*n* = 5). The average ± SD is shown from separate experiments. ***P < 0.001. (b) Downregulation of PHB inhibits expression of Bax and activation of caspase-3 and poly(ADP-ribose) polymerase (PARP) 9 h after ABR treatment as shown by western blot analysis.

Since ABR induced the expression of PHB, a tumor suppressor gene that may induce cell apoptosis by arresting the cell cycle at the G1/S phase, we focused on determining whether PHB participates in the apoptotic signaling triggered by ABR. Apoptosis induced by ABR (0.1 and 1 nM) was investigated using TUNEL method. As shown in Figure 3(a), group 1 and 2, the maximal apoptotic response was achieved 18 h after treating cells with ABR (77.7% apoptosis after 1 nM ABR treatment versus 38.5% apoptosis after 0.1 nM ABR treatment; *P* < 0.001). Although the specific PHB RNA interference (PHB siRNA) did significantly enhance cell apoptosis (PHB siRNA induces 22.8% apoptosis versus control siRNA induces 7.4% apoptosis, *P* < 0.001; Figure 3(a) groups 1 and 3), knockdown PHB reduced ABR-induced apoptosis (PHB siRNA reduced 1 nM ABR-induced apoptosis by 9.7%, *P* < 0.001, and PHB siRNA reduced 0.1 nM ABR-induced apoptosis by 16.6%, *P* < 0.001; Figure 3(a) groups 2 and 4). Apoptosis-related genes including Bax, caspase-3, and PARP were also examined. ABR significantly induced the expression of Bax (6.3-fold; *P* < 0.001) as well as the activation of caspase-3 and PARP in the Jurkat T cells (Figure 3(b), lanes 1 and 2). However, the activation was reduced when the PHB expression reduced (Figure 3(b), lanes 3 and 4). The data showed that PHB is involved in ABR-triggered apoptosis.

### 3.4. Upregulation of Human Bax Expression through Translocation of the PHB-p53 Complex from the Cytoplasm to the Nucleus

**Figure 4:**
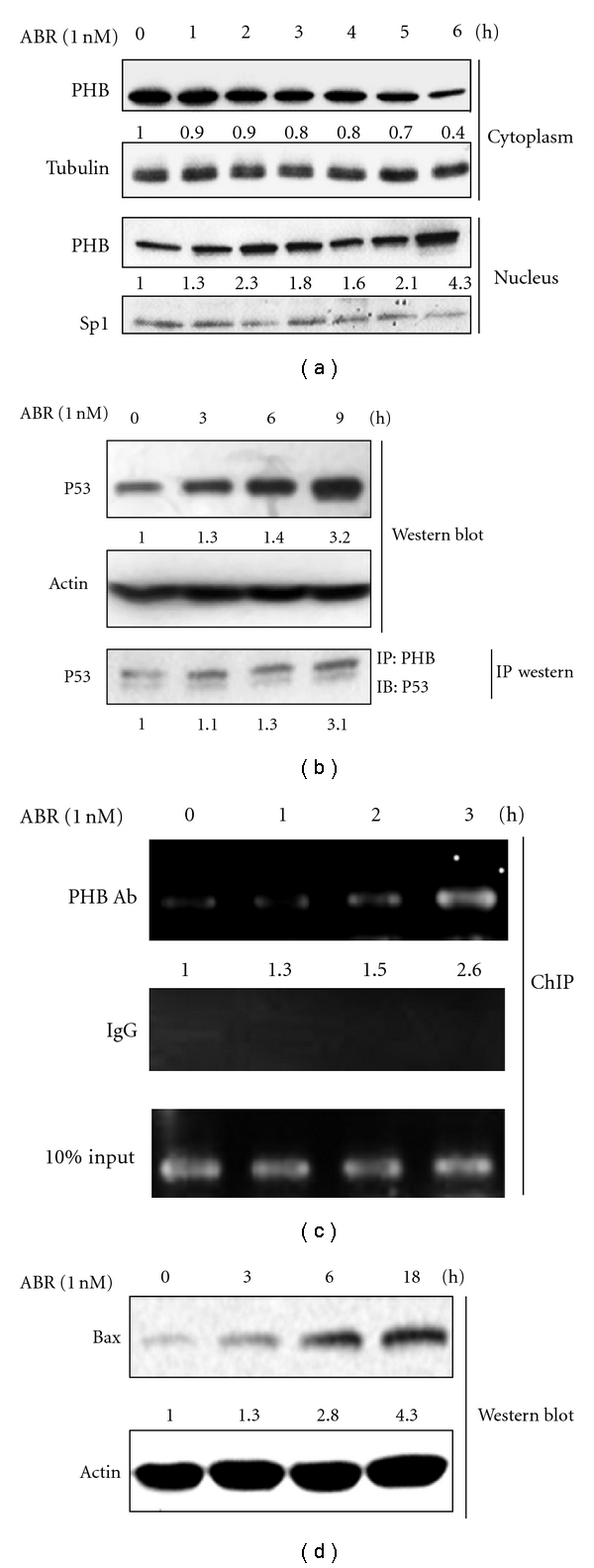
Prohibitin (PHB) induces the transcriptional activity of p53, which promotes expression of Bax. (a) Abrin (ABR)-induced translocation of PHB from cytoplasm to nucleus. (b) ABR upregulates p53 (western blot) and promotes the interaction between PHB and p53 in cells (immunoprecipitated western blot). (c) Association of PHB with the promoter region of the p53-targeted gene Bax.

The previously described results raised the possibility that PHB may be involved in the apoptotic processes triggered by ABR. On the other hand, increased levels of PHB in the nucleus may interact with the tumor suppressor protein p53 by which it exerts its apoptotic effect. Therefore, we attempted to determine whether there was an interaction between PHB and p53. As shown in Figure 4(a), PHB was translocated from the cytoplasm to the nucleus after ABR treatment for 6 h. Furthermore, a 1.3-, 1.4-, and 3.2-fold increase in p53 and a 1.1-, 1.3-, and 3.1-fold increase in the interaction with PHB were observed when cells were treated with ABR for 3, 6, and 9 h, respectively (Figure 4(b)). These results indicated that ABR may induce a physical interaction between PHB and p53 in the early stage of ABR-induced cell apoptosis. Since Bax is known to be one of the transcriptional regulation targets for PHB and p53, a ChIP assay was performed by using specific primers to amplify a potential p53-binding region in Bax. As shown in Figure 4(c), p53 was recruited to the promoter regions of Bax in a time-dependent manner. These results suggest that ABR induces the formation of the PHB-p53 complex in the nucleus, which enhances the transcriptional activity of p53 on Bax following apoptosis.

## 4. Discussion

Studies have shown that some proteins, including ABR, ricin, modeccin, diphtheria toxin, shiga toxin, and pseudomonas toxin, are apoptosis inducers [32–34]. Although ABR has been clearly identified as an inducer of apoptotic cell death by activating caspase-3 in several kinds of cancer cells [15, 35–38], the mechanisms of its involvement in cell apoptosis remain to be investigated. In this study, PHB is shown to be upregulated in a dose-dependent manner during ABR treatment and might play a potent role in ABR-triggered apoptosis by enhancing the activity and expression of p53. To the best of our knowledge, this is the first study to explain and demonstrate the role of PHB in ABR-induced apoptosis in human leukemia cells. The potential clinical applications of ABR may involve the enhancement of drug targeting as well as a decrease in side effects on noncancerous cells.

ABR is a RIP, which induces a shutdown of protein synthesis in target cells [33, 39]. However, previous reports also showed that the apoptosis-related protein Bax can be upregulated by ABR [40, 41]. Our results also indicated an overexpression of PHB and p53. One explanation is that cap-independent protein translation occurs in ABR-triggered apoptosis [42]. Hence, although PHB was first defined as a mitochondrial protein stabilizer [43], it was later shown to have diverse functions in a variety of processes including senescence, development, and tumor suppression [44]. In addition, PHB enhances the transcriptional activity of the tumor suppressor p53 via physical interaction [29, 45]. Our results are in agreement with earlier findings that PHB can interact with and upregulate p53 function during apoptosis [29]. It would, therefore, be interesting to determine the involvement of other coactivators in the PHB-p53 transcriptional activator complex upon ABR treatment.

Indeed, ABR may trigger cell apoptosis through its protein synthesis inhibition, ribotoxic stress, mitochondrial stress, PARP-induced NAD^+^ depletion, and ROS- and nuclease-induced DNA damage [46]. In addition, others and our previous works showed that ABR-induced apoptosis seems to occur either concomitant with or before the inhibition of protein synthesis [15, 46]. Although we have not yet determined a correlation between the 3 different ABR-induced pathways (including depurination activity, AOP-1 interaction, and prohibitin upregulation), both our current study and previous results indicated that either overexpression of AOP-1 or blockade of PHB expression may significantly reduce apoptosis (*P* < 0.05 for each). ABR upregulates the expression of, but does not interact with, PHB. On the contrary, ABR interacts with AOP-1 without upregulating it (data not shown). Although AOP-1 and PHB are thought to share several conserved domains that are expected to play similar roles in normal cells, the reason why they display distinct functions upon ABR treatment is still unclear; this would be an interesting topic for further studies. In addition, it seems that the interaction of ABR with AOP-1 is independent of depurination activity, and whether the upregulation of PHB depends on depurination is open to further study. Only an efficient cellular transport system for the toxicity-free mutant (ABR A chain E164Q) would be free of intact protein contamination (e.g., the reassociation of the A chain to the B chain). 

Moreover, our results here show that early growth response 1 (EGR1), a transcription factor that controls the early growth response and facilitates tissue healing, is significantly upregulated by ABR in leukemia cells. This result is in agreement with the response of lung epithelial cells to ricin [47], which may well serve as one of the markers of RIP-exerted toxicity. Ongoing studies are evaluating the potential of these ABR-related genes for clinical intervention. Nevertheless, as *Abrus precatorius* is labeled as a biological weapon which may be fatal if eaten, development of a passive vaccine or an antidote for ABR is necessary but under investigation [48, 49]. More understanding of the molecular mechanisms exerted by RIP family proteins may accelerate their clinical applications.

**Figure 5:**
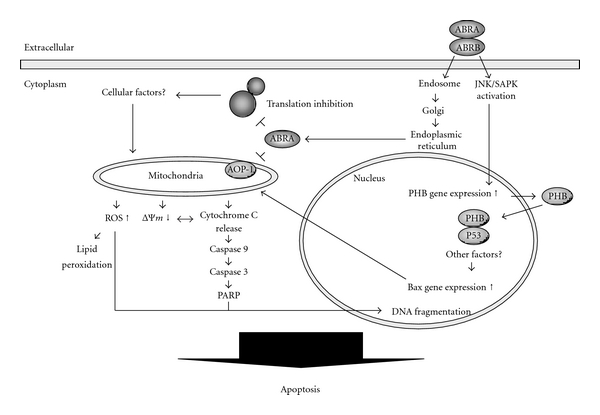
A model of abrin (ABR)-triggered apoptosis. ABR-induced apoptosis may occur through at least 3 pathways: first, inhibition of protein synthesis by its *N*-glycosidase activity; second, modulation of the function of mitochondria by specific interaction with antioxidant protein-1 (AOP-1); and third, interference with the transcription regulated by prohibitin (PHB). Repression of prohibitin attenuates ABR-triggered apoptosis via preventing the expression of BAX, cleaved-caspase 3, and cleaved-poly(ADP-ribose) polymerase (PARP). Once PHB is upregulated by ABR through the JNK/SAPK signaling pathway, the expression of proapoptotic gene Bax is turned on through the nuclear translocation and p53 interaction of PHB, by which activates the caspase cascade, and finally, apoptosis occurs.

In conclusion, we propose the model shown in Figure 5. ABR exhibits biological functions involving at least 3 pathways: translational inhibition, mitochondrial dysfunction, and transcriptional interfere through the upregulation of PHB. Since the downregulation of PHB significantly delays apoptosis induced by ABR, PHB could be employed in reducing the toxicity of immunotoxins and, hence, improve the efficiency of cancer chemotherapy.

## Figures and Tables

**Table 1:** Top 10 up-/downregulated genes changing in response to abrin exposure arranged by fold change. Gene common name, description, and gene ontology classification (where known) are listed.

Unigene number	Common name	Description	Fold change	GO biological process	GO molecular function	GO cellular process
Upregulation						

Hs.326035	EGR1	Early growth response 1	12.9	Transcription, DNA dependent	Transcription activator activity	Nucleus
Hs.502769	SLC3A2	Solute carrier family 3 (activators of dibasic and neutral amino acid transport), member 2	3.0	Transmembrane transport	Catalytic activity	Plasma membrane
Hs.2178	HIST2H2BE	Histone cluster 2, H2be	2.5	Nucleosome assembly	Binding to DNA and protein	Nucleus
Hs.82963	GNRH1	Gonadotropin-releasing hormone 1 (luteinizing-releasing hormone)	2.4	Multicellular organismal development	Hormone activity	Extracellular
Hs.467408	TRIM28	Tripartite motif-containing 28	2.3	Transcription, DNA dependent	Transcription coactivator/corepressor activity	Nucleus
Hs.514303	PHB	Prohibitin	2.3	Negative regulation of cell proliferation, gene-specific transcription from RNA polymerase II promoter by competitive promoter binding; regulation of apoptosis; signal transduction	Transcription activator/repressor activity	Cytoplasma, plasma membrane, mitochondria, nucleus
Hs.534404	RPL10	Ribosomal protein L10	2.2	Translation	Structural constituent of ribosome	Cytosol
Hs.5120	DYNLL1	Dynein, light chain, LC8-type 1	2.2	Induction of apoptosis	Motor activity	Cytosol
Hs.226390	RRM2	Ribonucleotide reductase M2	2.1	DNA replication	Oxidoreductase activity	Cytosol
Hs.202207	OSCP1	Organic solute carrier partner 1	2.1	Transport		Plasma membrane
Hs.25524	PTPN23	Protein tyrosine phosphatase, nonreceptor type 23	2.1	Cell projection organization	Hydrolase activity	Cytoplasma

Downregulation						

Hs.725987	TUBA1C	Tubulin, alpha 1c	−1.8	Cellular protein metabolic process	Structural molecule activity	Cytosol
Hs.535192	EEF1A1	Eukaryotic translation elongation factor 1 alpha 1	−1.6	Translation	Translation elongation factor activity	Cytosol
Hs.514581	ACTG1	Actin, gamma 1	−1.6	Cellular component movement	Protein binding	Cytosol
Hs.5662	GNB2L1	Guanine nucleotide-binding protein (G protein), beta polypeptide 2-like 1	−1.5	Positive regulation of apoptosis	Protein binding	Cytosol
Hs.534346	RPS7	Ribosomal protein S7	−1.5	Translation	Structural constituent of ribosome	Cytosol
Hs.433427	RPS17	Ribosomal protein S17	−1.5	Translation	Structural constituent of ribosome	Cytosol
Hs.5662	GNB2L1	Guanine nucleotide-binding protein (G protein), beta polypeptide 2-like 1	−1.5	Negative regulation of cell growth	Protein binding	Nucleus, cytosol
Hs.444467	EEF1G	Eukaryotic translation elongation factor 1 gamma	−1.5	Translational elongation	Translation elongation factor activity	Cytosol
Hs.514581	ACTG1	Actin, gamma 1	−1.5	Cellular component movement	Structural constituent of cytoskeleton	Cytosol
Hs.509736	HSP90AB1	Heat shock protein 90 kDa alpha (cytosolic), class B member 1	−1.5	Regulation of type I interferon-mediated signaling pathway	Unfolded protein binding	Cytosol
